# Responses of Dune Plant Communities to Continental Uplift from a Major Earthquake: Sudden Releases from Coastal Squeeze

**DOI:** 10.1371/journal.pone.0124334

**Published:** 2015-05-06

**Authors:** Iván F. Rodil, Eduardo Jaramillo, David M. Hubbard, Jenifer E. Dugan, Daniel Melnick, Carlos Velasquez

**Affiliations:** 1 Interdisciplinary Centre of Marine and Environmental Research (CIIMAR/CIMAR), University of Porto, Porto, Portugal; 2 Facultad de Ciencias, Universidad Austral de Chile, Valdivia, Chile; 3 Marine Science Institute, University of California Santa Barbara, Santa Barbara, California, United States of America; 4 Institut für Erd- und Umweltwissenschaften, Universität Potsdam, Postdam, Germany; University of New South Wales, AUSTRALIA

## Abstract

Vegetated dunes are recognized as important natural barriers that shelter inland ecosystems and coastlines suffering daily erosive impacts of the sea and extreme events, such as tsunamis. However, societal responses to erosion and shoreline retreat often result in man-made coastal defence structures that cover part of the intertidal and upper shore zones causing coastal squeeze and habitat loss, especially for upper shore biota, such as dune plants. Coseismic uplift of up to 2.0 m on the Peninsula de Arauco (South central Chile, ca. 37.5º S) caused by the 2010 Maule earthquake drastically modified the coastal landscape, including major increases in the width of uplifted beaches and the immediate conversion of mid to low sandy intertidal habitat to supralittoral sandy habitat above the reach of average tides and waves. To investigate the early stage responses in species richness, cover and across-shore distribution of the hitherto absent dune plants, we surveyed two formerly intertidal armoured sites and a nearby intertidal unarmoured site on a sandy beach located on the uplifted coast of Llico (Peninsula de Arauco) over two years. Almost 2 years after the 2010 earthquake, dune plants began to recruit, then rapidly grew and produced dune hummocks in the new upper beach habitats created by uplift at the three sites. Initial vegetation responses were very similar among sites. However, over the course of the study, the emerging vegetated dunes of the armoured sites suffered a slowdown in the development of the spatial distribution process, and remained impoverished in species richness and cover compared to the unarmoured site. Our results suggest that when released from the effects of coastal squeeze, vegetated dunes can recover without restoration actions. However, subsequent human activities and management of newly created beach and dune habitats can significantly alter the trajectory of vegetated dune development. Management that integrates the effects of natural and human induced disturbances, and promotes the development of dune vegetation as natural barriers can provide societal and conservation benefits in coastal ecosystems.

## Introduction

Ecological responses of coastal communities to natural or human-induced disturbances are influenced, in part, by the type and magnitude of the specific disturbance. Sandy beaches—the most prevalent coastal ecosystem along the ice-free coasts of the world [1]—are threatened by intense and transformative major disturbances, such as sea level rise and large storms, which cause coastal erosion and affect beach biota and ecosystem function [2–5]. Increasing coastal erosion, due to sea level rise, results in the restriction of coastal plant communities to a narrow upper-shore habitat zone [2,3]. Worldwide, the growing frequency and intensity of large storm events are also affecting coastal plant and macroinvertebrate communities through destabilizing habitat, increasing erosion and suppressing ecosystem recovery [4,5].

The construction of armouring structures, such as seawalls and rock revetments has been the most common societal response to threats from coastal erosion and shoreline retreat [6,7]. While plants of coastal dunes and strandlines are well-adapted to harsh physical conditions such as daily exposure to salt, winds, abrasion by sand and low nutrient availability [8,9], many of these plants seem unable to cope with the habitat loss and alteration associated with man-made structures, such as coastal defences. Coastal armouring alters beach and dune environments by directly covering habitat, constraining landward migration of the shoreline, and reflecting waves, encouraging continued erosion of the beach and leading to reductions in beach width [6,10,11]. As shown by recent field studies, as beach width narrows in front of armouring structures, habitat is eliminated from the upper and mid-intertidal zones resulting in loss of macroinfaunal diversity and ecosystem function [12–14]. The extent of coastal armouring can constrain the beach and upper shore community to such a narrow width that strandline plants are no longer able to establish or grow [5,15], resulting in upper-shores that are completely devoid of strandline vegetation, hummocks and dunes.

Coastal dune habitats support a valuable and dynamic plant community that can reduce the vulnerability of inland ecosystems to waves and wind. The relevance of the spatial distribution of dune vegetation as a major main factor controlling coastal vulnerability to storms has been recently recognized [16]. By acting as ecosystem engineers, dune vegetation may also influence the resiliency of coastal environments to climate fluctuations [16]. The spatial distribution of dune plants between the high tide line and the dune crest is a distinctive and highly dynamic phenomenon on open coast sandy beaches [17]. The zonation of coastal dune vegetation has been described for a number of regions worldwide [17–20]. However, there are no studies describing the responses of coastal dune vegetation to the combined effects of a major natural disturbance and coastal squeeze by armouring structures.

Catastrophic large-scale natural disturbances, such as earthquakes, tsunamis, volcanic eruptions, wildfires, hurricanes and floods, are key factors shaping natural ecosystems since they can change the state and the trajectory of an ecosystem [21–23]. Earthquakes, for instance, are extreme events occurring periodically along the seismically active coasts surrounding the Pacific Ocean. On 27 February 2010 (hereafter, 27F), a 500-km-long segment of the Nazca-South American plate boundary fault bordering the Chilean coast between ~ 34 and 38°S ruptured generating the Maule earthquake [24]. This seismic event reached a moment magnitude (M_w_) of 8.8, resulting in coseismic coastal land-level changes that reached a maximum uplift of 2.0 m in the Peninsula de Arauco (37.5° S), generating widespread damage and reshaping coastal landscapes [25–28]. Sandy beach ecosystems were affected by the devastating action of the tsunami associated with this earthquake as well as by the coastal uplift, with dramatic consequences for intertidal biota [13]. Coastal uplift associated with the Maule earthquake caused a dramatic increase of the total beach width up to 12.5 times in some places [13]. This extreme disturbance resulted in a significant expansion of the upper and mid-intertidal beach habitats potentially available for colonization by macrofaunal and plant communities.

In this study, we quantify the responses of coastal dune vegetation to habitat creation (i.e. increase in beach width) including the emergence of a new vegetated dune habitat following coseismic uplift using results obtained from ecological surveys conducted after the Maule earthquake (February 27, 2010). From 2012 to 2014 we surveyed dune vegetation on the uplifted sandy beach located in front of and adjacent to coastal armouring along the coast of Llico (Peninsula de Arauco, Chile; ca. 37.5° S). The recently emerged upper shore began to be colonized by dune-building plants nearly 21 months after the earthquake (November 2011), and subsequently a vegetated dune habitat started to take shape. The present study contributes with a thorough description of the initial development and zonation patterns of the main dune plant species from a very early stage on the earthquake and tsunami-affected coast of Llico. We also evaluated the effects of artificial coastal defence structures, and their interactions with an extreme event on these dune plants communities by comparing the spatial distribution of dune plants between armoured and unarmoured sites of the same beach.

## Materials and Methods

### Ethics statement

No specific permits were required for the described intertidal study sites. The sandy beach of Llico we studied in Chile is unrestricted to public access and use, and is not privately owned or designated as a protected area (reserve or park). The field study did not involve protected or endangered species.

### Study site and sampling design

**Fig 1:**
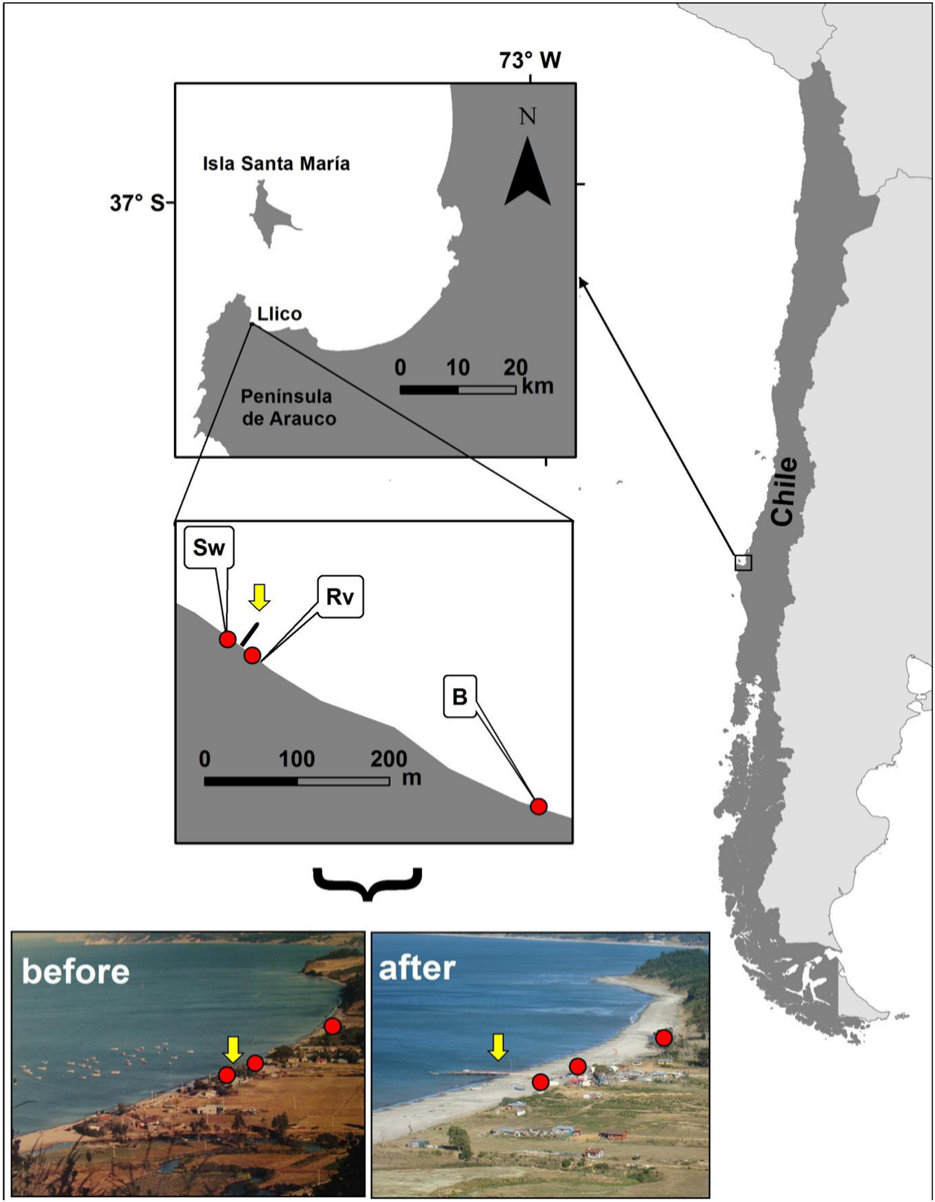
Location of the study sites at the beach of Llico, south central Chile. Before (July 2009) and after (December 2011) Maule earthquake (27^th^ February 2010) pictures of Llico are presented to show approximate locations (red dots) of sandy sites in front of the seawall (Sw) and the rocky revetment (Rv), as well as that of the unarmoured beach (B). Yellow arrows indicate location of a jetty in relation to low tide level. Note differences in intertidal width before and after the earthquake.

**Fig 2:**
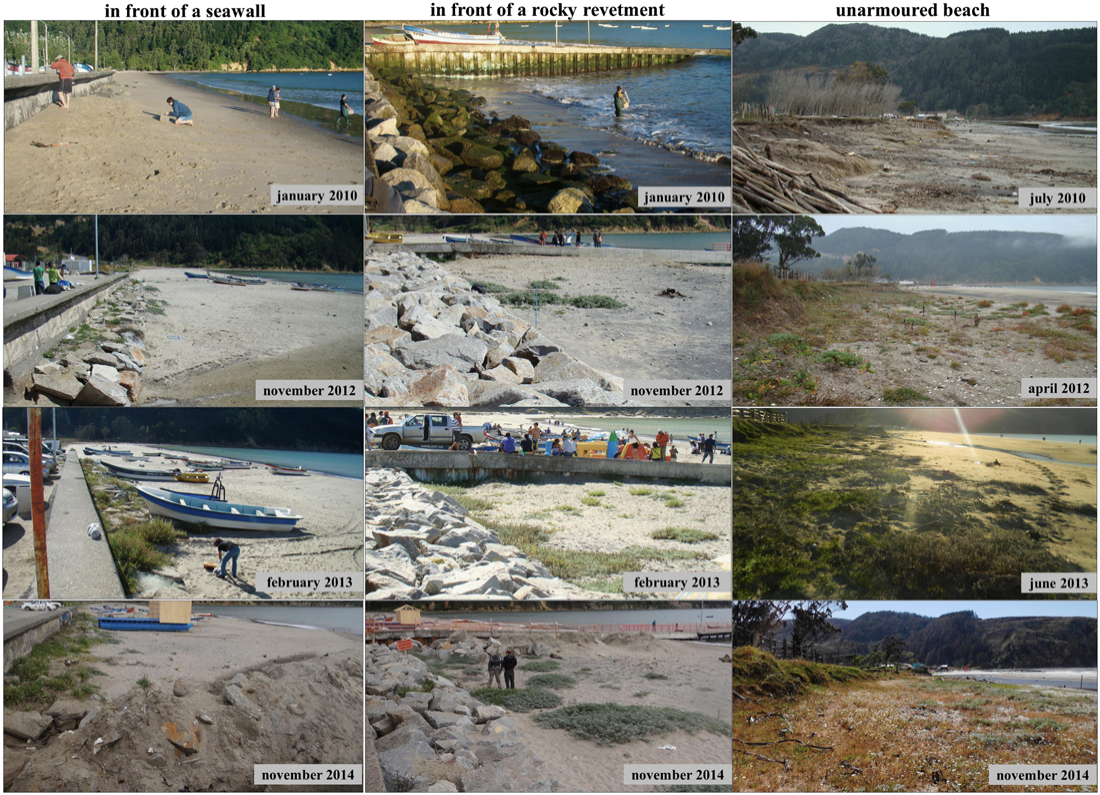
Before/after Maule earthquake (27th February 2010). Pictures of the armoured sites located in front of a seawall and a rocky revetment, and those of the unarmoured site taken at the beach of Llico at different stages of the study. Note the increasing human activities at the seawall site and the developed vegetation at the unarmoured site after the earthquake. Repair work on the concrete wall affected the vegetated zone at that site during November 2014.

This study was conducted at three sites located along the sandy beach of Llico (37°11’38” S, 73°33’44” W; Fig 1), on the northern coast of the Peninsula de Arauco in south central Chile. Continental uplift at this location during the Maule earthquake was estimated at around 2.0 ± 0.1 m by using a coralline alga [13], 2.0 ± 0.1 m by using an intertidal mussel [28], and 1.6 ± 0.05 m through GPS measurements at a geodetic benchmark [24]. The study area included two sites on armoured sections of the beach located on the southern and northern sides of a jetty used actively by fishermen for storing and launching boats; the site located north of the jetty (yellow arrow in Fig 1) is in front of a seawall while that located south is in front of a rocky revetment (Figs 1 and 2). The third study site was an unarmoured section of the beach located nearly 350 m north of the armoured sites (Figs 1 and 2). The three sites supported human activities and were subjected to frequent visitors, likely interfering with natural coastal vegetation dynamics (Figs 1 and 2). Although the degree of human interference appeared to be higher for the armoured sites than for the unarmoured site, no dune plants were observed on the upper shore levels of the three study sites in the year prior to the 2010 earthquake event (Figs 1 and 2).

Colonization of the upper shore levels of Llico sites by dune plants started most likely during November 2011 (~21 months after 27F). Data on dune plant communities was first collected during February 2012 (two years after the earthquake), followed by eight more sampling dates in 2012 (April, August and October), 2013 (January, June and November), and 2014 (January and November). Plant cover was measured on four shore-normal 50 cm-wide strip transects at each study site. The first transect was randomly placed perpendicular to the coast and the next three transects were spaced 5 m apart along the shore and fixed for the duration of the study. Transects extended across the entire distribution of vegetation: i.e. from the armoured structure (or the beginning of the sand) to the seaward limit of the vegetated area (total distance varied depending on the total width of each vegetated area). We estimated the cover of all the plants using 0.25 m^2^ (50 x 50 cm) quadrats placed contiguously along each transect. Visual estimates were made with the aid of 25 small squares (10 x 10 cm each) marked off within the quadrat frame, and percent cover of each plant species per quadrat was then calculated. Species were identified in the field, but when doubt existed, samples were collected to confirm identifications in the laboratory.

### Data analyses

The absolute cover of each plant species was calculated as the area of each species’ cover (m^2^) standardized by the total area sampled (m^2^) (*i*.*e*., absolute cover percentage expressed as a decimal number). We also estimated the amount of sandy substrate not occupied by plants.

Changes in width of the vegetated zone, number of plant species, sand cover, absolute total plant cover, and the absolute cover of the main plant species were analyzed separately through Generalized Linear Mixed Models (GLMMs) [29] with random slopes. Site (three levels: revetment, seawall, and beach) was considered a fixed factor, time (9 dates) a continuous co-variable, and random effects were associated with transects as a grouping factor. We assumed the dependent variable to be either Gaussian (width and cover), or Poisson distributed (number of plant species). We built different models considering the interaction of site and time and the random effects on both, the slope and the intercept (either correlated or not correlated), on the intercept alone, and finally on the slope alone. The most parsimonious model was selected according to AIC and through likelihood-ratio tests to formally compare the significance of the models. GLMMs were fitted by maximum likelihood assuming a Laplace approximation to the likelihood function. Model validity was checked by visual examination of residual plots and by assessment of dispersion statistics [29]. *A posteriori* contrasts were performed by reassigning the “Intercept” term sequentially to assess responses of individual levels of factors. All the computations were performed with the R software [30] and the “lme4” package [31].

The pooled data of the four transects at each site were used to plot kite diagrams (% cover) to describe zonation of plants over time. One-way analysis of similarity (ANOSIM) [31] on the basis of Bray-Curtis similarity matrices of absolute cover (4th-root transformed) for each plant species was used to test for differences in plant assemblages among sites over time (February, April, August, and October 2012, January, June, and November 2013, and January and November 2014). In addition, structure of plant assemblages over time in each site was visualized by non-metric multidimensional scaling (nMDS, see S1 Fig). A dummy species value of 1 was added to account for missing values (i.e., zero values in plots with no species present). Species that contributed most to the dissimilarities between sites and over time were identified using SIMPER analysis (see S1 Table). Multivariate data analyses were performed with PRIMER 6 [33].

## Results

Before the Maule earthquake (27 February 2010), the three study sites at Llico did not support dune or strand vegetation of any kind (Fig 2). Prior to the earthquake, both of the armouring structures occupied the upper shore levels and interacted directly with waves, even during lower tides (Fig 2); thus, no supratidal “dry sand” zones were present on these sites. The lack of an upper habitat at the armoured sites also greatly restricted human activities, including trampling, sunbathing, or the storage of boats on the upper beach and shoreline (Fig 2). After the coast was uplifted by 2.0 m, upper beach habitat suitable for these human activities became available at the armoured sites (Fig 2). In November 2014, part of the new vegetated area of the seawall site disappeared due to construction on the jetty (Fig 2). Prior to the earthquake, the upper shore habitats present on the unarmoured beach site also lacked dune or strand vegetation (Fig 2). Backed by a well-defined grassy area, this site provided dry sand areas subjected to recreational human uses. Two years after the first survey, the new upper beach habitat at the unarmoured site showed a more developed vegetated dune compared to the armoured sites (Fig 2).

### Analyses of the species richness in the vegetated zone

Dune plant communities at the three study sites were typically low in species richness (total of 10 species) (see S1 Table). Three of the dune plants observed were native species (*Nolana paradoxa* Lindl., *Rumex maricola* Remy, and *Schoenoplectus americanus* Pers.), while four were non-natives (*Ambrosia chamissonis* (Less.) Greene, *Cotula coronopifolia* L., *Matricaria chamomilla* L., and *Salsola kali* L.). Although with some controversy, the standard flora for Chile generally considers *C*. *coronopifolia* an introduced species [34]. Genus but no species was used for three plants (*Atriplex* spp., *Distichlis* spp. and *Hordeum* spp.).

**Fig 3:**
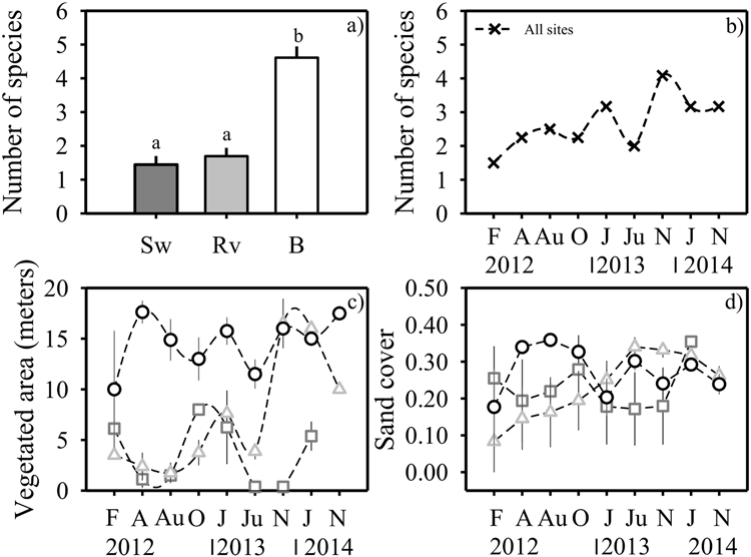
Responses of the plant species and vegetated area to the Maule earthquake. a) Mean (1 + SE, n = 4 transects) number of species at each site, b) total number of species including all sites, c) mean (1 ± SE, n = 4 transects) width of the vegetated area at each site, d) absolute sand cover (standardized mean ± SE, n = 4 transects) of the three study sites over time. The study sites at the beach of Llico are: Sw (in front of a seawall), Rv (in front of a revetment) and B (unarmoured beach). The sampling times were: February (F), April (A), August (Au) and October (O) 2012, January (J), June (Ju), and November (N) 2013, and January (J) and November (N) 2014.

Overall, the armoured sites showed the lowest values in species richness (up to six species) compared to the unarmoured site (up to nine species) (see S1 Table for species contribution, SIMPER). The number of plant species observed varied significantly among the three sites (Wald chisquare test: χ^2^ = 66.01, df = 2; p < 0.001). Thus, the seawall and revetment sites did not differ in species richness, but both differed significantly from the unarmoured beach site (Fig 3a, see Table 1 for pair-wise comparisons). The number of plant species increased significantly over time at all three sites (Wald test: χ^2^ = 18.9, df = 1; p < 0.001, Fig 3b).

**Table 1:** Results of GLMMs (model fit by maximum likelihood, Laplace approximation) containing pair-wise contrasts for the main response variables of the vegetated area at each study site.

Response variable	Contrast effects	Estimate	SE	z/t value	p
Number of species	B-Rv	-1.001	0.159	6.054	**<0.001**
	B-Sw	-0.983	0.160	-6.154	**<0.001**
	Rv-Sw	0.0184	0.18938	0.923	0.923
	Time	0.105	0.024	4.346	**<0.001**
	Transect (random effects): var = 0.002; SD = 0.043				
*full model*: *spp~Site+Time+(1|Transect)*, *family = poisson; AIC = 377*.*5*					
Vegetated area	B-Rv	-13.32	-2.182	-6.106	**<0.001**
(width)	B-Sw	-8.535	-2.182	-3.912	**<0.001**
	Rv-Sw	4.785	2.182	2.193	**<0.05**
	B*Ti—Rv*Ti	1.2	0.388	3.096	**<0.01**
	B*Ti—Sw*Ti	-0.563	0.388	-1.451	0.150
	Rv*Ti—Sw*Ti	-1.763	0.388	-4.547	**<0.001**
	Transect (random effects): var = 1.25; SD = 1.12				
*full model*: width~Site*Time+(1|Transect), *family = gaussian; AIC = 624*.*97*					
Sand	B-Rv	-0.1956	0.0646	-3.028	**<0.01**
	B-Sw	-0.0588	0.0825	-0.714	0.512
	Rv-Sw	0.1368	0.0664	2.059	**<0.05**
	B*Ti—Rv*Ti	0.0305	0.0113	2.701	**<0.01**
	B*Ti—Sw*Ti	-0.0026	0.0139	-0.189	0.858
	Rv*Ti—Sw*Ti	-0.0331	0.0115	-2.212	0.087
	Transect (random effects): var = 0.01; SD = 0.09				
*full model*: sand~Site*Time+(Site*Time|Transect), *family = gaussian; AIC = -89*.*6*					
Total plant cover	B-Rv	-0.0179	0.018	-0.998	0.320
	B-Sw	-0.043	0.018	-2.403	**<0.05**
	Rv-Sw	-0.025	0.018	-1.405	0.163
	Time	0.003	0.0028	1.228	0.222
	Transect (random effects): var = 7.7 10e^-4^; SD = 0.03				
*full model*: *cover~Site+Time+(1|Transect)*, *family = poisson; AIC = 225*.*34*					

Models included site (B: unarmoured beach, Rv: revetment and Sw: seawall) as a fixed factor, time (9 dates) as a covariate, and random effects were associated with transects as a grouping factor. The best fitted full model is indicated per response variable. SE: standard error; var: variance; SD: standard deviation; AIC: Akaike’s information criterion. z values for models with Poisson error structures, t values for models with Gaussian error structures. Significant effects appear in bold.

### Analyses of the plant cover in the vegetated zone

The width of the vegetated zone varied significantly among sites (see Table 1 for pair-wise comparisons). In general, the vegetated zone at the unarmoured beach site was significantly wider than the zones at the armoured sites (Fig 3c), but this pattern was not consistent over time (i.e., significant site*time interaction, Wald test: χ^2^ = 21.5, df = 2; p<0.001). The width of the vegetated zone in front of the revetment was significantly greater than in front of the seawall in the last sampling dates (Fig 3c, Table 1). Also, this width differed significantly between the beach and revetment sites at the beginning of the surveys, and between the beach and seawall sites for all the sampling dates (Fig 3c, Table 1).

The amount of bare, unvegetated sand varied significantly among sites, but this pattern was not consistent over time (i.e. significant site*time interaction, χ^2^ = 8.5, df = 2; p<0.05). The revetment site showed an increase in the cover of bare sand in the dune zone over time, and the unarmoured beach site showed a higher proportion of bare sand at the beginning of the surveys (2012) (Fig 3d, Table 1). The cover of bare sand at the beach also differed significantly from that of the seawall site over time (Fig 3d, Table 1).

**Fig 4:**
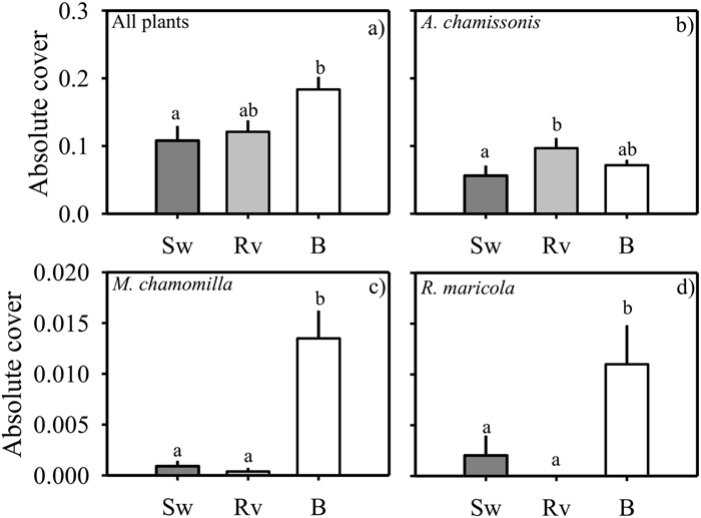
Absolute cover of a) all plants, b) *Ambrosia chamissonis*, c) *Matricaria chamomilla*, and d) *Rumex maricola*. The plots show the standardized mean values (1 + SE, n = 4 transects) at the three study sites (Sw: seawall, Rv: revetment and B: unarmoured beach).

**Fig 5:**
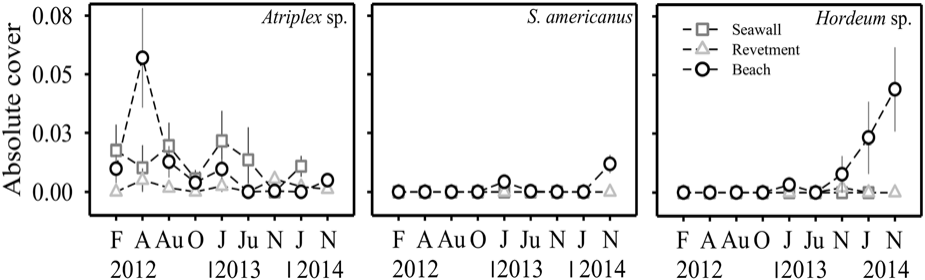
Absolute cover of *Atriplex* spp., *Schoenoplectus americanus*, and *Hordeum* spp. The plots show the standardized mean values (1 ± SE, n = 4 transects) of the three study sites over time (sampling times and study sites as in Fig 3).

The total cover of dune vegetation was always significantly higher (χ^2^ = 5.8, df = 2; p<0.05) at the unarmoured beach compared to the armoured sites (Fig 4a, Table 1). In general, six species comprised most of the vegetation cover in all the sites (S1 Table). The cover of three of those species, *A*. *chamissonis* (χ^2^ = 5.8, df = 2; p<0.05), *M*. *chamomilla* (χ^2^ = 43.9, df = 2; p<0.001), and *R*. *maricola* (χ^2^ = 11.1, df = 2; p<0.01) showed significant differences among sites. The cover of *A*. *chamissonis* was significantly greater at the revetment than at the seawall sites, and no significant differences were found between the revetment and unarmoured sites (Fig 4b, see Table 2 for pair-wise comparisons). The cover of *M*. *chamomilla* and *R*. *maricola* were significantly greater at the unarmoured than at the armoured sites (Fig 4c–4d, Table 2). Three plants, *Atriplex* spp. (χ^2^ = 7.56; p<0.05), *S*. *americanus* (χ^2^ = 16.2; p<0.001) and *Hordeum* spp. (χ^2^ = 29.3; p<0.001) showed significant interactions (i.e. site*time, df = 2 for all tests, Table 2). As a result, the cover of *Atriplex* spp. at the unarmoured site was significantly greater than at the revetment site in April 2012 (Fig 5). For *Atriplex* spp., significant differences were also found between the seawall and the revetment sites, but this pattern was not consistent over time (Table 2, Fig 5). The cover of both *S*. *americanus* and *Hordeum* spp. was significantly greater at the unarmoured than at the armoured sites in the later sampling dates (2014) (Fig 5).

**Table 2:** Results of GLMMs (model fit by maximum likelihood, Laplace approximation) containing pair-wise contrasts for the main plant species (standardized absolute cover, see Methods) of the vegetated area at each study site.

Response variable	Contrast effects	Estimate	SE	t value	p
*A*. *chamissonis*	Be-Rv	0.0210	0.0157	1.34	0.185
	Be-Sw	-0.0168	0.0157	-1.068	0.288
	Rv-Sw	-0.0378	0.016	-2.404	**<0.05**
	Time	0.0042	0.003	1.70	0.093
	Transect (random effects): var = 5.2e^-4^, SD = 0.023				
*full model*: *Ambrosia~Site+Time+(1|Transect)*, *family = gaussian; AIC = 241*.*35*					
*M*. *chamomilla*	Be-Rv	-0.0131	0.0022	-5.853	**<0.001**
	Be-Sw	-0.0126	0.0022	-5.623	**<0.001**
	Rv-Sw	0.0005	0.0022	0.231	0.818
	Time	-0.0002	0.0003	-0.518	0.602
	Transect (random effects): var = 7.2e^-6^, SD = 0.003				
*full model*: *Matricaria~Site+Time+(1|Transect)*, *family = gaussian; AIC = -686*.*2*					
*R*. *maricola*	Be-Rv	-0.0110	0.0035	-3.12	**<0.01**
	Be-Sw	-0.0090	0.0035	-2.55	**< 0.01**
	Rv-Sw	0.0000	0.0006	0	1
	Time	0.0004	0.0006	0.741	0.460
	Transect (random effects): var = 0, SD = 0				
*full model*: *Rumex~Site+Time+(1|Transect)*, *family = gaussian; AIC = -593*.*44*					
*Atriplex* spp.	Be-Rv	-0.028	0.008	-3.582	**<0.001**
	Be-Sw	-0.007	0.0077	-0.904	0.368
	Rv-Sw	0.0206	0.0077	2.678	**<0.01**
	Be*Ti—Rv*Ti	0.0037	0.0014	2.72	**<0.001**
	Be*Ti—Sw*Ti	0.0014	0.0014	1.028	0.306
	Rv*Ti—Sw*Ti	-0.0023217	0.00137	-1.694	0.0933
	Transect (random effects): var = 4.7e^-6^, SD = 0.002				
*full model*: Atriplex~Site*Time+(1|Transect), *family = gaussian; AIC = -589*.*01*					
*S*. *americanus*	Be-Rv	0.0129	0.0052	2.496	**<0.05**
	Be-Sw	0.0130	0.0051	2.52	**< 0.05**
	Rv-Sw	0.000	0.005	-0.02	0.982
	Be*Ti—Rv*Ti	-0.0042771	0.0009	4.65	**<0.001**
	Be*Ti—Sw*Ti	-0.0043	0.001	-4.73	**<0.001**
	Rv*Ti—Sw*Ti	0.0001	0.0009	0.076	0.940
	Transect (random effects):var = 6.6e^-7^, SD = 0.001				
*full model*: Scoenoplectus~Site*Time+(1|Transect), *family = gaussian; AIC = -676*.*16*					
*Hordeum* spp.	Be-Rv	0.0022	0.0013	1.68	0.0968
	Be-Sw	0.0022	0.0013	1.68	0.097
	Rv-Sw	0.0000	0.001	0	1
	Be*Ti—Rv*Ti	-0.0008	0.0002	-3.49	**<0.001**
	Be*Ti—Sw*Ti	-0.0008	0.0002	-3.49	**<0.001**
	Rv*Ti—Sw*Ti	0.0000	0.0002	0	1
	Transect (random effects): var = 0, SD = 0				
*full model*: Hordeum~Site*Time+(1|Transect), *family = gaussian; AIC = -976*					

Models included site (B: unarmoured beach, Rv: revetment and Sw: seawall) as a fixed factor, time (9 dates) as a covariate, and random effects were associated with transects as a grouping factor. The best fitted full model is indicated per response variable. SE: standard error; var: variance; SD: standard deviation; AIC: Akaike’s information criterion. Significant effects appear in bold.

### Zonation and composition of dune plant assemblages

**Fig 6:**
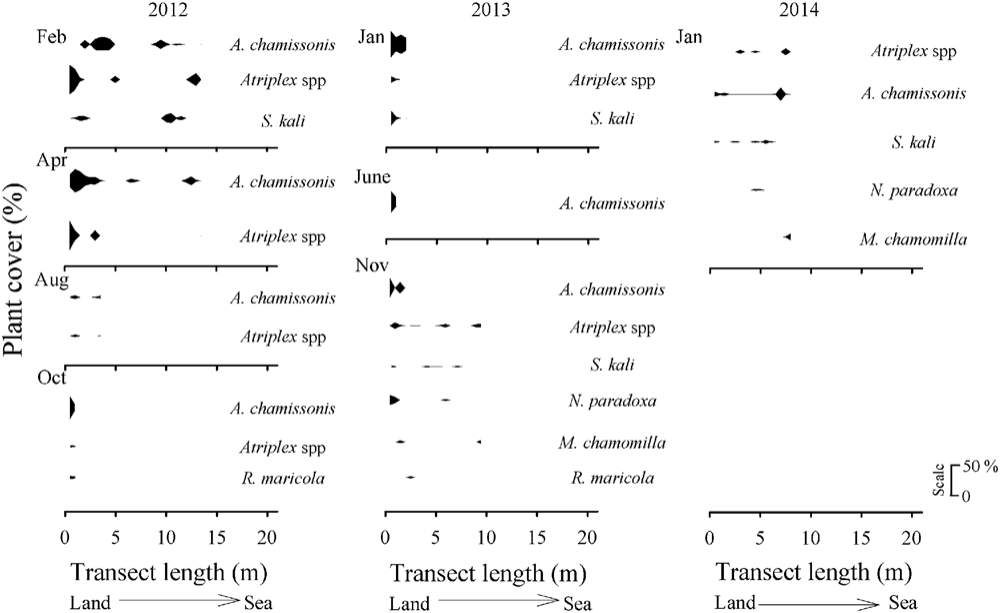
Across-shore distribution over time of the dune plant species (plant cover in %). Sandy beach site located in front of the seawall at Llico (scale: 0–50%). Upper-shore levels started at 0 m. The sampling times were: February, April, August, October 2012 January, June, November 2013, and January 2014. Repair work on the concrete wall affected the vegetated zone at this site during November 2014; thus, no sampling was carried out at that month.

**Fig 7:**
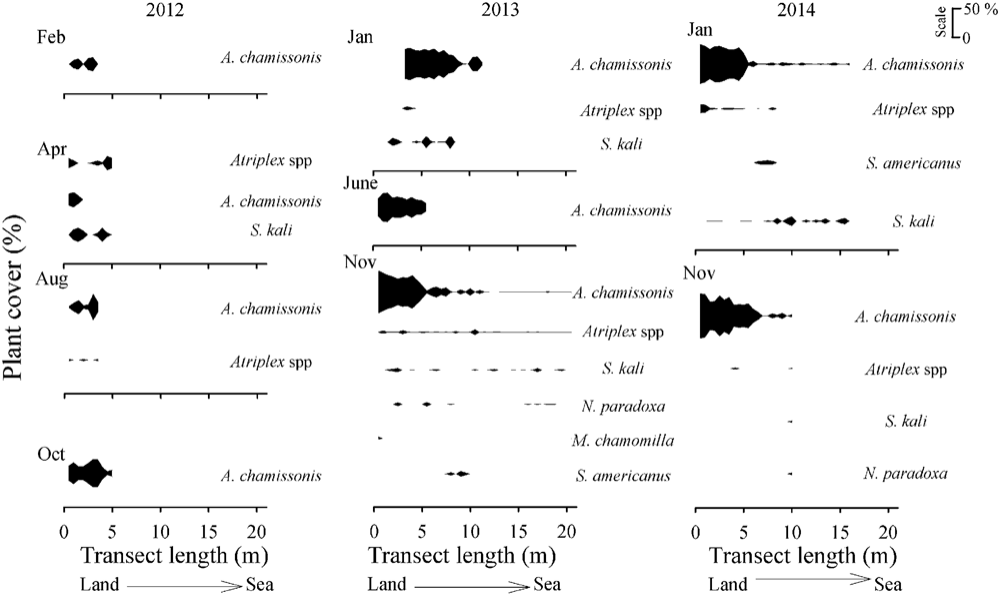
Across-shore distribution (m) over time of the dune plant species (plant cover in %). Sandy beach site located in front of the rocky revetment at Llico (scale: 0–50%). Upper-shore levels started at 0 m. The sampling times were: February, April, August, October 2012 January, June, November 2013, and January, November 2014.

Total plant cover was higher at the upper shore levels, especially at the site with the seawall (Fig 6). The most common plant species at the armoured sites were *A*. *chamissonis*, *Atriplex* spp. and *S*. *kali* (S1 Table). The native species *R*. *maricola* was recorded at the upper shore of the seawall site in October 2012 (<10%, on average), and then again in November 2013 (< 1%, Fig 6). *N*. *paradoxa* and *M*. *chamomilla* were recorded in the last sampling dates at the mid and upper levels of the seawall and revetment sites (Figs 6 and 7). The sedge, *S*. *americanus*, was found in the mid level of the vegetated zone at the revetment for the first time in November 2013, and again in the next survey in January 2014 (< 10%, Fig 7).

**Fig 8:**
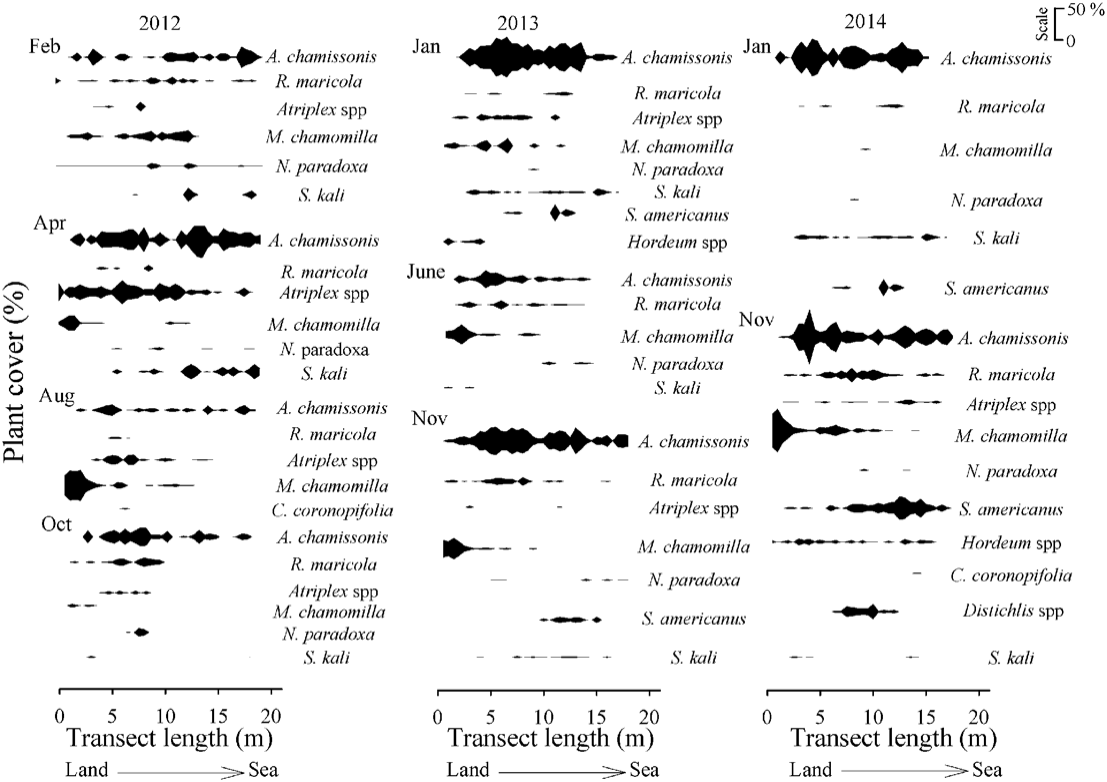
Across-shore distribution (m) over time of the dune plant species (plant cover in %). Located at the unarmoured sandy beach site at Llico (scale: 0–50%). Upper-shore levels started at 0 m. The sampling times were: February, April, August, October 2012 January, June, November 2013, and January, November 2014.

At the unarmoured site, six species (*A*. *chamissonis*, *Atriplex* spp., *M*. *chamomilla*, *N*. *paradoxa*, *R*. *maricola* and *S*. *kali)* were recorded on all sampling dates (S1 Table). Three species, *A*. *chamissonis* (5–30%), *Atriplex* spp. (1–13%), and *R*. *maricola* (1–6%), extended throughout the whole vegetated zone over time (Fig 8). *M*. *chamomilla* was recorded at the upper-shore levels, while *N*. *paradoxa* and *S*. *kali* were observed in the mid-low levels of the vegetated zone (Fig 8). *C*. *coronopifolia* was recorded in August 2012 at the upper levels, and again in November 2014 (~ 1%). The sedge, *S*. *americanus* was counted at the mid vegetated zone in the last sampling dates in 2014 (Fig 8). For grasses, *Hordeum* sp was recorded at the upper part of the dune in June 2013 (< 5%) and November 2014 (~ 3%), and *Distichlis* spp. was recorded in November 2014 (< 5%) (Fig 8).

**Fig 9:**
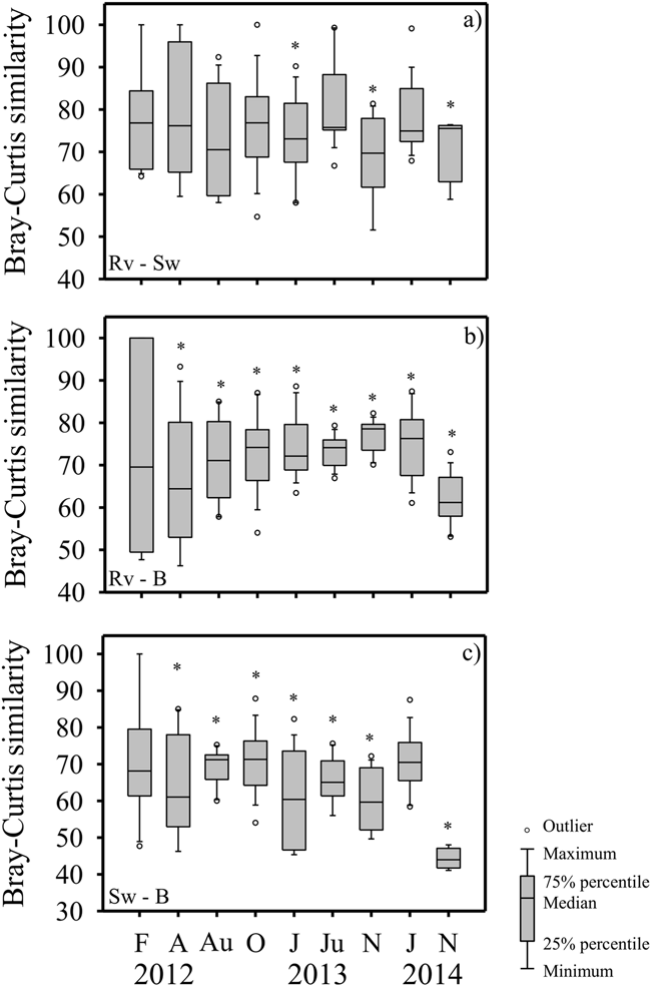
Similarity in the dune plant assemblages among the beach sites over time. Similarity of plant assemblages (note scale: 40–100%) between a) Revetment—Sewall, b) Revetment—Beach, c) Sewall—Beach were calculated from the Bray-Curtis similarity matrix (ANOSIM, S2 Table). Sampling times as in Fig 3. *significant differences (*p* < 0.05) in the similarity of plant assemblages between sites (Rv *vs* Sw, Rv *vs* B, and Sw *vs* B) at each time.

ANOSIM indicated that the absolute cover of the dune plant assemblages changed significantly among the three sites over time (S2 Table), illustrated through the ordination plots and summary (Figs 9 and S1). During the first survey in February 2012 no significant differences were detected among the 3 sites, but over time significant differences among sites developed and persisted (Fig 9). Since April 2012 significant differences in the similarity of plant assemblages were found between unarmoured and armoured sites (Fig 9). Dune plant assemblages were similar between the revetment and seawall sites, although significant differences between these two armoured sites were found in January and November 2013, and then again in November 2014 (Fig 9, S2 Table).

## Discussion

On 27 February 2010, the Chilean coast between ~ 34 and 38°S was affected by the Maule earthquake, resulting in widespread damage and coastal land-level changes largely affecting intertidal landscapes in the region. Our results provided striking new evidence of the rapid colonization of dune plants on one sandy beach after such an extreme event, and the subsequent development of vegetated dunes in the previously absent high-shore habitats created by the (2 m) coseismic uplift. However, the consequences of interactions between the uplift (which resulted in beach widening), the presence of man-made armouring structures, and the new opportunities for tourism and fishing activities provided by the new habitat complicated the responses of dune plant communities at three sites of Llico beach. We found that plant community structures associated with the early colonization and zonation of the new vegetated dune habitat were initially quite similar among the study sites, but that they diverged significantly between the armoured and the unarmoured sites over the course of our study.

Before the earthquake, the presence of defence structures in the two armoured sites prevented the development of any upper shore vegetated zones, and also restricted human uses of the beach such as sunbathing or storing of boats at these sites. Armouring had also eliminated populations of common high-intertidal wrack-associated macroinvertebrates at these sites [13] as shown elsewhere [12,35, 36] where armouring structures are a common feature of the littoral landscape. Prior to this extreme event, the upper shore habitat present on the unarmoured site was subjected to human uses, such as sunbathing and trampling, which can interfere with the natural dynamics of dune and strandline vegetation [37,38]. Nonetheless, this site supported populations of high-intertidal wrack-associated macroinvertebrates before and after the earthquake [13].

During the earthquake, continental uplift strongly affected all the study sites, transforming intertidal habitats at the armoured sites into upper shore “dry sand” habitats, where dune plant species emerged and thrived within a relatively short period of time. The coastal dune zonation and temporal patterns of vegetation across the beach sites showed that plants colonized the newly created habitat in less than two years after which, dunes and hummocks developed quickly. This result suggests that the lack of plants seaward of armoured sites prior to the earthquake was largely due to the constraint of the man-made defences on coastal evolution. Thus, the post-earthquake creation of a sandy upper shore habitat in front of the coastal defences at Llico, had a significant and positive effect on dune and strandline vegetation.

Other upper-beach biota, in particular the wrack-associated invertebrates, exhibited positive post-earthquake uplift responses at the armoured sites at this beach [13]. However, the contrast in the timing of the appearance of wrack-associated invertebrates (<2 months, [13]) and vegetation (21 months, this study) in the new sandy habitats created by coseismic uplift on armoured beach is striking. This contrast suggests that while mobile wrack-associated invertebrates persisted and were readily available at the adjacent unarmoured site to colonize the new uplifted habitats, the lack of a local seed bank may have inhibited the recruitment of plants at all sites. The size of seed banks in coastal dunes on a barrier island scaled with the frequency of disturbance and the time since disturbance, as well as successional state, with poorly developed and more transient seed banks found in frequently disturbed coastal habitats [39]. Local seed banks also appeared to be extremely reduced on groomed beaches in southern California, resulting in negligible recruitment of coastal strand vegetation [40]. In the present study, a combination of coastal armouring and human uses prior to the 2010 earthquake may have reduced the local seed bank and delayed recruitment of plants until sufficient seeds were delivered from source areas via marine processes. In addition, debris left by the tsunami may have also covered some of the habitat suitable for plant colonization in front of the seawall site until it was removed in late 2011 (see Fig 2 in [13]).

The coseismic uplift of the coast was a clear opportunity to allow natural processes to create vegetated strand and dune communities that could store sand, provide shelter from erosion, enhance biodiversity and become self-sustaining coastal landscape features. This process began at a similar time at the three study sites, but after a few months it slowed significantly at the armoured sites. Following the earthquake and the coseismic uplift, the plant community of the unarmoured beach site showed the highest diversity and plant cover throughout the entire course of our two year study. This coastal evolution included well-developed hummocks covered by a diverse plant community including upland plants, such as *M*. *chamomilla* or *S*. *americanus* at very early stages of succession, and salt tolerant grass species such as *Distichlis* spp. These contrasting results for armoured and unarmoured sites may represent and indirect rather than a direct effect of the armouring, which already existed before the earthquake and coastal uplift [13]. Man-made artificial defences, such as those as Llico, are built not only to protect coastal sites from erosion and retreat, but also to facilitate human shoreline uses, such as fishing and tourism, vital to local economies. Widespread activities, including strolling [37], mechanical raking [40], vehicle use [41], and the landing and storing of boats (as illustrated in our study) can result in habitat disturbances that retard the evolution of coastal dune vegetation. Following the earthquake these, likely new, disturbances were frequently observed at the armoured sites of Llico (especially in front of the seawall). Also the level of human activity may have declined at the unarmoured site after the earthquake due to the availability of ample new upper dry sand beach habitats for recreational use proximal to the armouring structures and coastal access point. For this reason, we hypothesize that the result of lower number of plant species, diversity and plant cover observed at the armoured sites over the course of the study, as compared to the unarmoured site, was related to the new human uses of these formerly intertidal sites. Our results and observations suggest that human activities slowed down the plant colonization and dune development at the armoured sites, as compared to the unarmoured site. Nonetheless, at the three sites, the development of perennial vegetation trapped wind-blown sand, and generated embryonic dunes that transformed the uplifted coastal habitats, providing an important ecotone between intertidal and terrestrial ecosystems in a coastal area that had lacked this fundamental natural transition zone and barrier before the earthquake.

A well-developed and diverse dune plant community acts as an ecosystem engineer that provides unique ecosystem function and services, including buffering intertidal habitats and protecting sensitive inland ecosystems from inundation [42]. However, disturbance-induced shifts in species richness, composition and zonation, as observed at the armoured sites in this study, could result in reduced sand-holding ability and reduced dune-building capacity to buffer and respond to coastal erosion from waves and storms in the future [2,3,16]. Recent major earthquakes and tsunamis demonstrate the vulnerability of highly populated shorelines to these large-scale events and the role of natural ecosystems, such as dunes and forests, as buffers for human infrastructure [42,43]. In the wake of a major natural disaster, our results illustrate how the development of new coastal dune vegetation, that can serve as a natural barrier against erosion by ocean forces, can be significantly affected by interactions between coastal armouring and human activities.

Patterns of colonization and zonation by dune plants on the uplifted upper shore at other sites along the coast affected by the Maule earthquake were broadly similar to those we observed at Llico. However, as shown in our results from Llico, it is difficult to generalize these responses across sites, even at a single beach location, due to the variety of human uses and intensities. Nonetheless our study illustrates how natural experiments can be useful tools to understand the consequences of unpredictable large disturbance events on coastal ecosystems [5,13,43]. Our findings have important implications for coastal management and emphasize the need to understand the ecological responses to major natural events on coastal ecosystems, and their interactions with human interventions and activities [2,5,13,44,45]. This is not trivial, since the use of coastal armouring is expected to increase in the near future given the likelihood of sea-level rise and continued erosion of the majority of the world’s coastlines [2,11,12,36], the cyclical occurrence of catastrophic events [46–48], and the exponential growth of human populations in coastal areas [49]. Our study represents a unique example of the need to develop strategies and coastal management approaches that effectively integrate effects of natural and human induced disturbances while promoting the development of dynamic strand and dune vegetation to provide benefits for both society and conservation in vulnerable coastal ecosystems.

## Supporting Information

S1 Fig Graphic results of non-metric multidimensional scaling (nMDS) analyses. Plots showing differences in dune plant species assemblages (normalized absolute cover) among the three sandy beach sites studied over time.
(TIF)

S1 Table Absolute cover of the main plant species. Table shows species names, indication of the species average (% cover standardized and expressed as a decimal number), and contribution to the similarity per site and over time (SIMPER).
(DOC)

S2 Table Results for the one way analysis of similarity (ANOSIM). Data showing dissimilarities (R, statistical correlation coefficient) in the absolute plant cover (standardized mean values) of the dune plant assemblages among Llico sites: unarmoured beach (B), revetment (Rv), and seawall (Sw) over time. R: gives the strength of the similarity between sites (0–1). R values close to 1 indicate high dissimilarity between sites, and values close to 0 indicate no dissimilarity between sites. Significant results in italics.
(DOC)
