# Supplementary figures and images for: Responses of Dune Plant Communities to Continental Uplift from a Major Earthquake: Sudden Releases from Coastal Squeeze
Source: PLoS One. 2015 May 6;10(5):e0124334. doi: 10.1371/journal.pone.0124334 (PMC4422612; doi:10.1371/journal.pone.0124334)

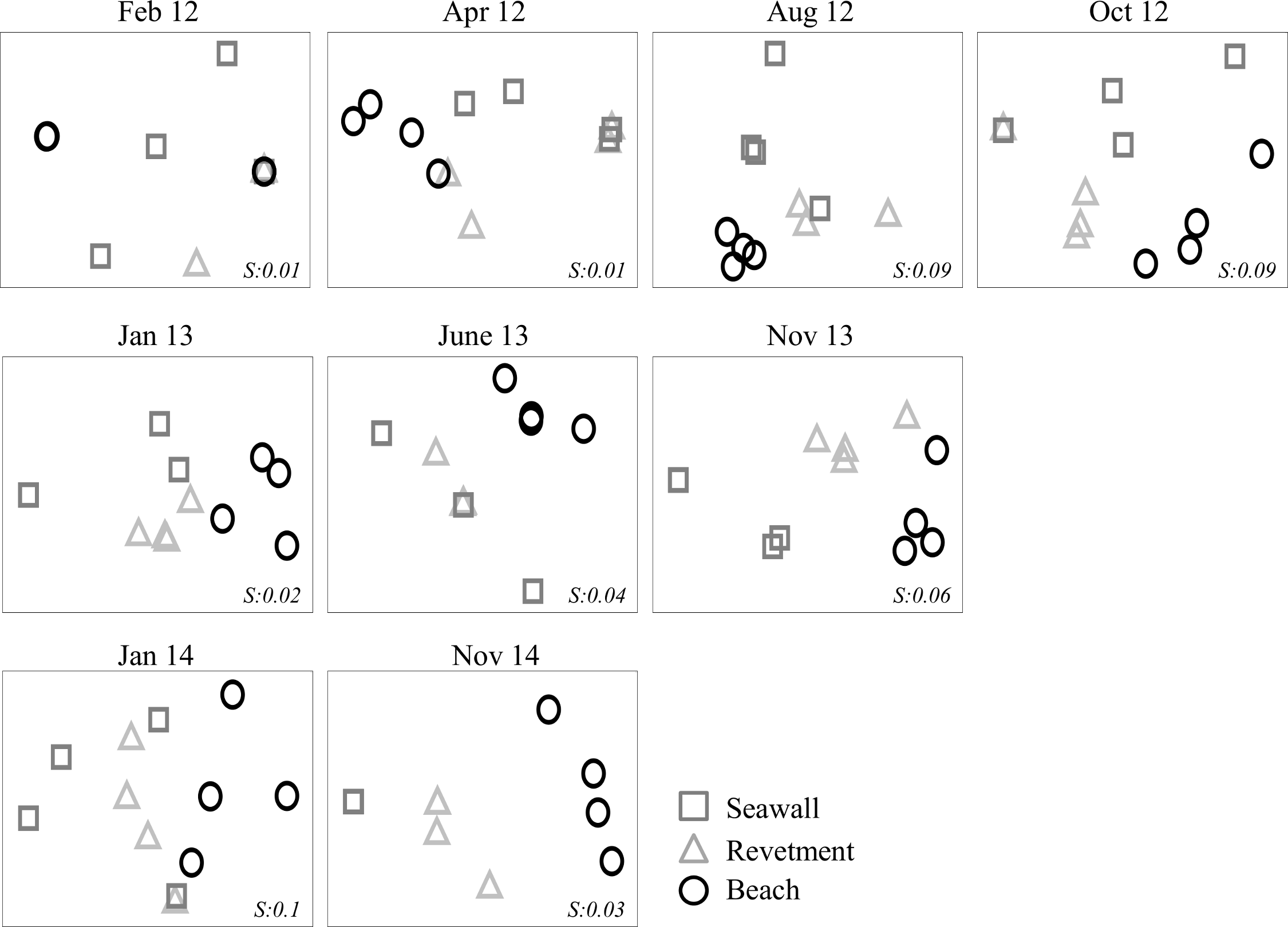

Supplement: S1 Fig — Plots showing differences in dune plant species assemblages (normalized absolute cover) among the three sandy beach sites studied over time. (TIF) [file pone.0124334.s001.tif]
