# Supplementary material for: Responses of Dune Plant Communities to Continental Uplift from a Major Earthquake: Sudden Releases from Coastal Squeeze
Source: PLoS One. 2015 May 6;10(5):e0124334. doi: 10.1371/journal.pone.0124334 (PMC4422612; doi:10.1371/journal.pone.0124334)
Supplement: S1 Table — Table shows species names, indication of the species average (% cover standardized and expressed as a decimal number), and contribution to the similarity per site and over time (SIMPER). (DOC) [file pone.0124334.s002.doc]

| Site | Species | Cover | Similarity | Contribution (%) | Cumulative (%) |
| --- | --- | --- | --- | --- | --- |
| Revetment | *Ambrosia chamissonis* | 0.44 | 41.59 | 76.53 | 76.53 |
|  | *Salsola kahli* | 0.13 | 7.65 | 14.08 | 90.62 |
| Seawall | *Ambrosia chamissonis* | 0.28 | 16.07 | 59.82 | 59.82 |
|  | *Atriplex* sp. | 0.16 | 6.84 | 25.45 | 85.27 |
|  | *Salsola kahli* | 0.1 | 3.14 | 11.67 | 96.94 |
| Beach | *Ambrosia chamissonis* | 0.48 | 28.28 | 41.65 | 41.65 |
|  | *Matricaria chamomilla* | 0.26 | 11.91 | 17.53 | 59.18 |
|  | *Atriplex* sp. | 0.2 | 8.87 | 13.07 | 72.25 |
|  | *Rumex maricola* | 0.21 | 7.63 | 11.24 | 83.49 |
|  | *Salsola kali* | 0.18 | 5.88 | 8.67 | 92.15 |
|  | - | - | - | - | - |
| Time | Species | Relative Cover | Similarity | Contribution (%) | Cumulative (%) |
| Feb-12 | *Ambrosia chamissonis* | 0.22 | 4.94 | 35.61 | 73.71 |
|  | *Atriplex* sp. | 0.13 | 5.28 | 38.11 | 38.11 |
|  | *Matricaria chamomilla* | 0.07 | 1.01 | 7.32 | 81.03 |
|  | *Nolana paradoxa* | 0.05 | 0.93 | 6.73 | 94.89 |
|  | *Salsola kali* | 0.1 | 0.99 | 7.14 | 88.16 |
| Apr-12 | *Ambrosia chamissonis* | 0.33 | 15.2 | 39.36 | 39.36 |
|  | *Atriplex* sp. | 0.24 | 10.52 | 27.25 | 66.61 |
|  | *Salsola kahli* | 0.21 | 9.32 | 24.12 | 90.73 |
| Aug-12 | *Ambrosia chamissonis* | 0.39 | 24.01 | 43.63 | 43.63 |
|  | *Atriplex* sp. | 0.24 | 13.5 | 24.53 | 68.16 |
|  | *Matricaria chamomilla* | 0.14 | 11.95 | 21.71 | 89.87 |
|  | *Salsola kahli* | 0.12 | 5.57 | 10.13 | 100 |
| Oct-12 | *Ambrosia chamissonis* | 0.38 | 30.95 | 69.22 | 69.22 |
|  | *Rumex maricola* | 0.11 | 7.45 | 16.67 | 85.89 |
|  | *Atriplex* sp. | 0.12 | 4.29 | 9.58 | 95.48 |
| Jan-13 | *Ambrosia chamissonis* | 0.47 | 28.04 | 46.91 | 46.91 |
|  | *Salsola kahli* | 0.27 | 17.2 | 28.77 | 75.68 |
|  | *Atriplex* sp. | 0.2 | 6.35 | 10.63 | 86.3 |
| Jun-13 | *Ambrosia chamissonis* | 0.46 | 44.02 | 77.4 | 77.4 |
|  | *Matricaria chamomilla* | 0.11 | 8.1 | 14.24 | 91.64 |
| Nov-13 | *Ambrosia chamissonis* | 0.47 | 28.54 | 47.06 | 47.06 |
|  | *Atriplex* sp. | 0.13 | 8.72 | 14.38 | 76.49 |
|  | *Salsola kahli* | 0.17 | 9.12 | 15.04 | 62.1 |
|  | *Matricaria chamomilla* | 0.12 | 4.95 | 8.16 | 93.61 |
|  | *Rumex maricola* | 0.11 | 5.43 | 8.96 | 85.45 |
| Jan-14 | *Ambrosia chamissonis* | 0.48 | 39.86 | 70.52 | 70.52 |
|  | *Atriplex* sp. | 0.14 | 6.4 | 11.32 | 81.84 |
|  | *Salsola kahli* | 0.15 | 3.27 | 5.79 | 87.63 |
|  | *Rumex maricola* | 0.09 | 2.83 | 5.01 | 92.64 |
| Nov-14 | *Ambrosia chamissonis* | 0.38 | 44.88 | 62.62 | 62.62 |
|  | Scirpus americanus | 0.14 | 6.23 | 8.69 | 71.31 |
|  | *Hordeum* sp | 0.11 | 5.78 | 8.06 | 79.37 |
|  | *Atriplex* sp. | 0.1 | 4.14 | 5.77 | 85.15 |
|  | *Matricaria chamomilla* | 0.09 | 3.75 | 5.23 | 90.38 |
