# Supplementary material for: Responses of Dune Plant Communities to Continental Uplift from a Major Earthquake: Sudden Releases from Coastal Squeeze
Source: PLoS One. 2015 May 6;10(5):e0124334. doi: 10.1371/journal.pone.0124334 (PMC4422612; doi:10.1371/journal.pone.0124334)
Supplement: S2 Table — Data showing dissimilarities (R, statistical correlation coefficient) in the absolute plant cover (standardized mean values) of the dune plant assemblages among Llico sites: unarmoured beach (B), revetment (Rv), and seawall (Sw) over time. R: gives the strength of the similarity between sites (0–1). R values close to 1 indicate high dissimilarity between sites, and values close to 0 indicate no dissimilarity between sites. Significant results in italics. (DOC) [file pone.0124334.s003.doc]

|  | 2012 | | | |  | 2013 | | |  | 2014 | |
| --- | --- | --- | --- | --- | --- | --- | --- | --- | --- | --- | --- |
|  | February | April | August | October |  | January | June | November |  | January | November |
| Global R test | 0.04 | *0.389^*^* | *0.567^**^* | *0.403^**^* |  | *0.569^**^* | *0.537^**^* | *0.773^**^* |  | 0.236 | *0.914^**^* |
| Sites |  |  |  |  |  |  |  |  |  |  |  |
| Rv - Sw | 0.078 | -0.083 | 0.25 | 0.083 |  | *0.438^*^* | *0.156* | *0.729^*^* |  | -0.042 | *0.9^*^* |
| Rv -B | 0.052 | *0.51^*^* | *0.667^*^* | *0.615^*^* |  | *0.802^*^* | *0.979^*^* | *0.781^*^* |  | *0.406^*^* | *0.9^*^* |
| Sw - B | -0.115 | *0.698^*^* | *0.833^*^* | *0.5^*^* |  | *0.656^*^* | *0.625^*^* | *0.99^*^* |  | 0.354 | *0.9^*^* |

^*^significant differences (*p* < 0.05); ^**^highly significant differences (*p* < 0.01).
